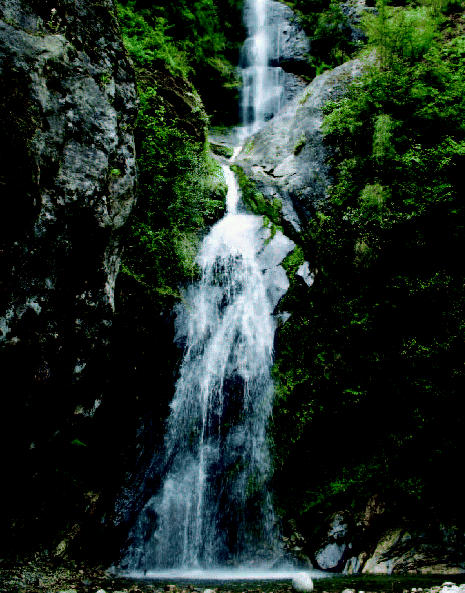# The Beat

**Published:** 2006-12

**Authors:** Erin E. Dooley

## Debugging Without Methyl Bromide

Food and farming uses are among the few applications still allowed for the pesticide methyl bromide, which has been largely phased out under the Montréal Protocol. Now engineers at the University of California, Davis, have developed a new sterilization method for fresh produce that could replace post-harvest needs for this ozone-depleting chemical. The new method, metabolic stress disinfection, subjects pests to alternating vacuum and carbon dioxide treatments, which effectively suffocates the insects. Although the initial cost of the equipment is higher than methyl bromide, the chemicals used in the process are much cheaper. The gases used also can be recovered and recycled.

**Figure:**
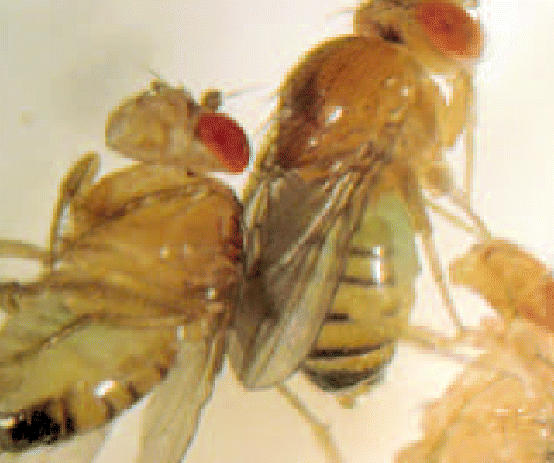


## EPA PM Standard Standoff

Internal documents from the EPA show that the agency could be protecting more lives each year if it had adopted stringent particulate air pollution standards advocated by an agency advisory panel. The documents reveal that 5,000–10,000 Americans could be saved from air pollution–related strokes, heart disease, and lung disease with stronger standards; instead, the agency opted in September 2006 to retain existing particulate rules. In a press release, the EPA said that the standards as set are still the most protective in history. The panelists disagree, however, and on 29 September 2006 sent EPA administrator Stephen Johnson a letter stating that “there is clear and convincing scientific evidence” that significant adverse human health effects occur at the current standards.

## The Biomonitor

On 29 September 2006 California governor Arnold Schwarzenegger enacted a statewide program, one that is both voluntary and confidential, to measure levels of chemical contaminants in people. The program, the first of its kind in the United States, could be in place by 2007. Program data will make up a statewide report on environmental chemical exposures, and smaller studies on communities of concern and statewide trends will be conducted in future years as needed. Another program goal is to prioritize chemicals for study. A nine-member scientific panel appointed by Schwarzenegger and the state legislature will guide the program.

**Figure:**
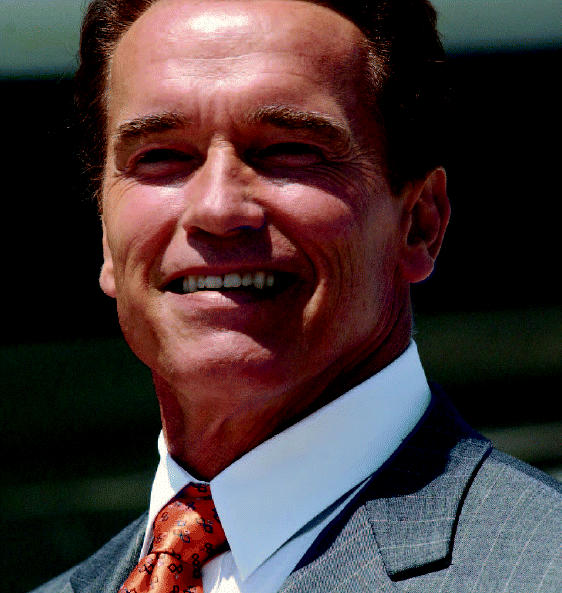


## Musician Spreads Word About Water

UN statistics show that nearly 2 million children die each year because of unclean water supplies and poor sanitation. Now popular musician Jay-Z is set to star in a documentary titled *Diary of Jay-Z: Water for Life*, part of a larger educational program on global issues sponsored by the music channel MTV. The documentary will be shot during Jay-Z’s world tour, when he will be visiting areas in Turkey, South Africa, and other countries without access to safe drinking water. The program will also feature projects that are working on sustainable solutions to the problem, and will call on viewers to take action by helping abroad and at home. MTV and the UN will augment the program with a special website, free classroom access to the documentary, and supporting lesson plans.

**Figure:**
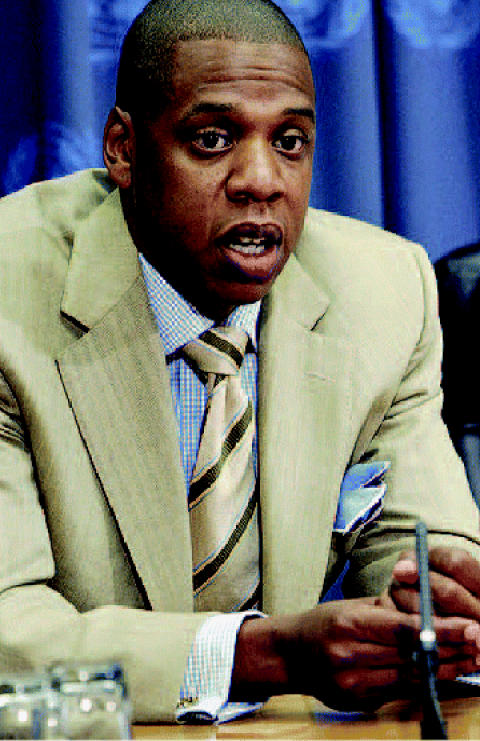


## Inventorying Emissions in Mexico

September 2006 witnessed the publication of Mexico’s first national inventory of atmospheric air emissions, which was produced with support from the Commission for Environmental Cooperation and U.S. partners. The inventory provides detailed data on nitrogen oxides, sulfur dioxide, volatile organic compounds, carbon monoxide, ammonia, and particulate matter for each Mexican state and municipality. The report covers industrial, mobile, and natural sources of these pollutants. The inventory, part of a five-year effort to standardize air emissions reporting across North America, is a key step toward improving air quality management both within Mexico and on each side of its borders.

## New Hope for Nepal’s Environment

Over the past decade, ongoing civil war between Maoist and government forces in Nepal has disrupted many conservation activities in the country. A ceasefire was declared in May 2006, however, and a month later the parties agreed to establish a new interim government and draft constitution. At this point, advocacy groups including WWF Nepal, CARE Nepal, and the IUCN drew up a set of recommendations for preserving biodiversity, forest areas, and reserve lands in the country, which the constitutional committee agreed to incorporate into the draft constitution. Among other recommendations are a call to keep up to 40% of Nepal’s land under forest cover in perpetuity, and to provide for investments in conservation and sustainable use of areas of biodiversity.

**Figure:**